# A Dedicated Genetic Algorithm for Localization of Moving Magnetic Objects

**DOI:** 10.3390/s150923788

**Published:** 2015-09-18

**Authors:** Roger Alimi, Eyal Weiss, Tsuriel Ram-Cohen, Nir Geron, Idan Yogev

**Affiliations:** Applied Physics Division, Soreq NRC, Yavneh 81800, Israel; E-Mails: eyal_we@soreq.gov.il (E.W.); tsuriel@soreq.gov.il (T.R.-C.); nirg@soreq.gov.il (N.G.); idan_yogev@amat.com (I.Y.)

**Keywords:** genetic algorithms, magnetic dipoles, magnetic sensors, tracking problems

## Abstract

A dedicated Genetic Algorithm (*GA*) has been developed to localize the trajectory of ferromagnetic moving objects within a bounded perimeter. Localization of moving ferromagnetic objects is an important tool because it can be employed *in situ*ations when the object is obscured. This work is innovative for two main reasons: first, the *GA* has been tuned to provide an accurate and fast solution to the inverse magnetic field equations problem. Second, the algorithm has been successfully tested using real-life experimental data. Very accurate trajectory localization estimations were obtained over a wide range of scenarios.

## 1. Introduction

Magnetic sensor arrays are commonly used to estimate the location of moving ferromagnetic objects. A ferromagnetic object creates an anomaly in the natural earth magnetic field due to its high magnetic permeability compared to non-ferromagnetic materials. This anomaly takes the form of a distortion of the signal that can be detected by an array of dedicated magnetic sensors. The measurements may be processed by an estimation algorithm in order to determine the object’s trajectory and its magnetic moment. The estimation of a ferromagnetic object location can be used in many applications. For example, in traffic control systems magnetic induction sensors are used to estimate the presence of a vehicle [1]. Magnetic systems are widely used to estimate the location of unexploded mines [2]. In the cold war, magnetic systems were used to detect submarines [3]. Magnetic systems can also be used to detect concealed ferromagnetic objects carried by people inspected in a security check point [4].

A conventional deterministic approach based on the Levenberg-Marquard Algorithm (*LMA*) [5,6] can be used to estimate location. The main advantage of the *LMA* is its rapidity and its well-known and reliable implementation. However, the results provided by the *LMA* are typically satisfactory only when it is provided with a relatively good initial guess and when the signal to noise ratio (*SNR*) is larger than five. Moreover, *LMA* does not provide a quality index score that allows accepting or rejecting the numerical outcome. 

Among conventional meta-heuristics schemes, like Particle Swarm Optimization, Simulated Annealing, Gravitational search algorithm, we have tested the Ant Colony Optimization (*ACO*) [7]. Besides its complex implementation, the main drawbacks of *ACO* scheme are the computing time required for convergence and the low precision of the solution.

Conventional localization techniques are not applicable in many magnetic localization systems because they require either high *SNR* or time consuming computing processes. In this work, we propose an adapted genetic algorithm especially tuned for localization of a moving ferromagnetic object. The drawbacks of the *LMA* were resolved by adapting a Genetic Algorithm (*GA)* to the magnetic localization problem where the initial guess issue is resolved by tailoring a suitable sampling of the initial search-domain. The scheme works well even for *SNR* close to one. The confidence value provides an excellent quality index for estimating the reliability of the solution.

### Genetic Algorithms

Genetic Algorithms are a family of computational models inspired by evolution [8,9,10]. The concept of the algorithms is to encode potential solutions on a simple chromosome-like structure and to apply recombination operators in order to preserve critical genes. It is applying the principal concept “survival of the fittest” on genes that achieves best results. Genetic algorithms are often viewed as function optimizers although the range of problems to which genetic algorithms have been applied is quite broad. 

An implementation of a genetic algorithm begins with creating a population of random chromosomes. Then, reproductive opportunities are planned in a way that favors chromosomes, which yield a better fitting solution to the problem. The better fitting chromosomes are given better chances to reproduce than those chromosomes, which yield a poorer solution. Mutation operators are applied in order to guarantee a good exploration of the search domain.

Genetic algorithms are used in a wide variety of applications, for example, data mining and data analysis [11], electrical engineering and circuit design [12], image processing [13], planning and scheduling [14], chemistry and chemical engineering [15,16], geometry and physics [17,18], economics and finance [19,20], medicine [21,22,23], networking and communication [24,25], and control systems design [26,27,28].

Since the pioneer work of Wynn on ferromagnetic objects tracking [29] several studies have been performed that process the magnetic data recorded by sensor arrays using genetic algorithms [30,31]. 

The basic scheme of the *GA* as usually employed in most of the references above cannot be used in many practical real-time applications. There are two main reasons for that: running time and precision of the result. Typical convergence times may range from several minutes to several hours in real-life conditions. In real-time applications this is unacceptable. A few second response time is usually required using a standard PC platform, which is a great challenge for *GA* calculation. Furthermore, the required accuracy of the localization of the moving object is in the order of a few percent only. This is usually only possible by running multiple parallel initial population sets and defining a selection criterion to pick the best result among all the different solutions.

The present work is innovative in two main features: first the *GA* has been upgraded to improve accuracy and efficiency. Second, the algorithm has been applied to real experimental data and not only in a clean simulation or in a sterile laboratory scenario. 

## 2. Experimental Section

**Figure 1:**
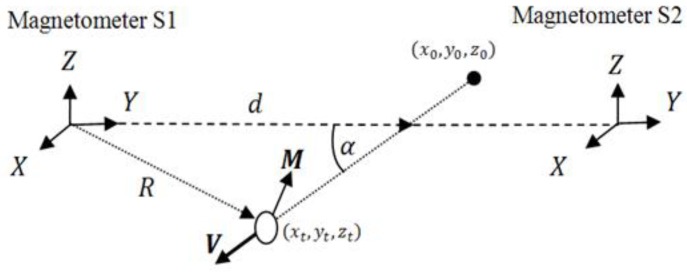
The experimental setup comprises two vector magnetometers that measure the change in magnetic flux generated by the motion of a ferromagnetic object (see solid line) along a straight line at a constant velocity.

The experimental setup consists of a two tree-axial fluxgate sensors array (Mag634 made by Bartington instruments Inc., Witney, England) fixed to the ground (see Figure 1). From a strict mathematical point of view, at least two sensors are required since there are six unknowns: the three position coordinates and the three moment components, and each sensor provide three equations. The sensor output is sampled by a 16 bit NI 6225 data acquisition module at a 10 Hz rate. In Figure 1, *d* and *α* represent the crossing point and the crossing angle, respectively. *V* and *M* denote the velocity and moment vectors, respectively, and *R* is the dynamic distance between the moving object and the magnetic sensor. The time dependent coordinates of the ferromagnetic object are denoted by *x_t_, y_t_* and *z_t_*.

The ferromagnetic objects are free to move about the sensor array. The measurement environment is clear of magnetic noise and clutter. The object motion is at a constant velocity and along a straight line and can cross the sensor line at any point or angle. The objects linear velocity is a typical walking velocity of around 1 m/s.

## 3. Genetic Algorithm Description

### 3.1. Data Preprocessing

As stated above the core engine of the tracking procedure is of a standard Genetic Algorithm. However, in order for a *GA* to run efficiently and to converge rapidly, pre-processing of the data is required. This is done in two steps:

The experimental data is filtered and detrended (*i.e.*, slow trend and *DC* removal). The filter employed is a low-pass Discrete Cosine Transform (*DCT*) [32] that smoothens the data and efficiently removes the high frequency noise. For the detrending, *i.e.*, removal of *DC* shift and linear drifts, we have adopted the Hodrick-Prescott filter [33]. It is based on assessing the smoothness prior to approach and operates like a time varying finite impulse response (FIR) high-pass filter. It has been shown that the filter can be written in a matrix form using the expression:

(1) $$S_{f}=\;\left(I-{\left(I+\lambda^{2}D_{2}^{T}D_{2}\right)}^{-1}\right)S$$

where *S* is the signal before detrend, *S_f_* is the signal after detrend, *I* is the *N* by *N* identity matrix (*N* is the number of samples), *λ* is a smoothing parameter (typically of the order of 10,000) and *D*_2_ is the *N* by (*N* − 2) second order differencing matrix of the form:

(2) $$D_{2}=\left(\begin{array}{cccccc} 1 & -2 & 1 & 0 & \cdots & 0\\ 0 & 1 & -2 & 1 & \ddots & \vdots \\ \vdots & \ddots & \ddots & \ddots & \ddots & 0\\ 0 & \cdots & 0 & 1 & -2 & 1 \end{array}\right)$$

After smoothing and detrending, a relevant portion of the data is selected for *GA* analysis. For this purpose, the total field (the norm of the three axes of the sensors) of each signal is calculated and the center positions (denoted by *C_1_* and *C_2_*) and width *σ_1_* and *σ_2_* of each curve are estimated using normal distribution analysis. Only data points in the range between *C_1_* − 1.5*σ_1_* and *C_2_* + 1.5*σ_2_* are used. The resulting signal length to be processed by the *GA* may vary from 100 to 200 sample points depending on the object velocity distance from the sensors.

### 3.2. Initial Population

An initial population of chromosomes is randomly selected. Each chromosome contains six genes that when plugged into the magnetic flux equations produce a three-dimensional linear trajectory on which the field measured by the two sensors can be calculated. The genes are: the crossing angle *α* at which the trajectory crosses the sensors (expressed in degrees °), the crossing distance *d* (m) which is the point where the two lines cross and the first sensor, the three components of the magnetic moment of the moving object *M* (Am^2^) on the *X*-axis (noted *M_x_*), on the *Y*-axis (*M_y_*) and on the *Z*-axis (*M_z_*), and the velocity *v* (m/s). The domain spanned by these variables will be referred to as the search-domain of the *GA*. For each gene the initial values are uniformly sorted within a given range. Each chromosome uniquely defines a 3D path [*x*(*t*) *y*(*t*) *z*(*t*)] where *z*(*t*) is taken as a constant height above ground, along which the magnetic fields recorded by the two sensors can be calculated according to the relation

(3) $$\left|\begin{array}{c} B_{x}\\ B_{y}\\ B_{z} \end{array}\right|=\frac{\mu_{0}}{4\pi R^{5}}\left|\begin{array}{ccc} 3x^{2}-R^{2} & 3xy & 3xz\\ 3yx & 3y^{2}-R^{2} & 3yz\\ 3zx & 3zy & 3z^{2}-R^{2} \end{array}\right|\left|\begin{array}{c} M_{x}\\ M_{y}\\ M_{z} \end{array}\right|$$

where *x = x*(*t*) *− x_sensor_*, *y = y*(*t*) *− y_sensor_*, *z = z*(*t*) *− z_sensor_*, where *x_sensor_*, *y_sensor_* and *z_sensor_* denote the sensor coordinates and *R* is the distance between the point on the path and the sensor. A “reproductive” chromosome is a chromosome that generates magnetic fields that “fit” (*i.e.*, are similar to) the ones recorded by the sensors. Since the quantitative evaluation of the fitness (similarity) is a key issue of the genetic algorithm, it is discussed in detail in the next section.

### 3.3. Fitness Calculation

It is well known [34] that the selection of the fitness criteria is critical for the performance of genetic algorithms, both in the quality of the result and in the convergence speed. Optimal fitness functions must fulfill two criteria:

First, the fitness should be sensitive enough to provide reliable information regarding how well the calculated field reproduces the recorded data. In other words, it should be a steep function of the variables that are of physical importance. In our case the crossing angle and position, the direction of motion and the total magnetic moment of the object. Steepness assures rapid convergence of the algorithm. However, over-sensitivity may lead to unstable behavior, since every small change may shift the output away from the solution we are looking for. A good trade-off is usually provided by complex functions of the searching variables. 

Second, since the fitness is typically evaluated many times during the main *GA* loop, its calculation must be simple enough to provide low *CPU* (Computer Process Unit) time cost. Obviously complex functions are not easily implemented and require long computation times, therefore the two criteria often contradict. Choosing the optimal fitness according to these criteria is well illustrated in the present study. 

Comparing the calculated and the recorded fields quantitatively gages the similarity between the two sets of curves (six pairs). One of the most rigorous topological approaches is provided by the Fréchet distance [35]. 

A popular definition of the Fréchet distance between two curves is the minimum length of a leash required to connect a dog and its owner as they walk without backtracking along their respective curves from one endpoint to the other. The Fréchet distance, although very accurate, is difficult to implement. It has been shown [36] that for the type of curves we are estimating, the Hausdorff distance is a close alternative to the Fréchet distance. It is less precise but much easier to compute. The Hausdorff distance is the maximum distance of a curve to the nearest point in the other curve. Two curves are close in the Hausdorff distance if every point of either curve is close to some point of the other set. If *P* and *Q* are the curves we compare, then the Hausdorff distance is given by

(4) $$\delta \left(P,Q\right)=\text{max}\left(\tilde{\delta }\left(P,Q\right),\tilde{\delta }\left(Q,P\right)\right)$$

where

(5) $$\tilde{\delta }\left(P,Q\right)\;=\;\underset{X\in P}{\max}\underset{Y\in Q}{\min}\left||X-Y|\right|$$

where ||.|| denotes the *L_2_* norm (Euclidian distance). Since the Hausdorff distance is defined on [0,∞], and because it is more convenient for the fitness *f_h_* to be defined on [0,1], we apply the following transformation:

(6) $$f_{h}\left(P,Q\right)=\text{exp}\left(-\delta \left(P,Q\right)\right)$$

In our case *P* is the experimental curve (magnetic data recorded by the sensor) and *Q* is the curve calculated using the genes carried by a given chromosome (geometric path and magnetic moment). 

Another and much simpler way to evaluate the proximity of the graphs of two 2D functions is to consider how well the graphs superpose, that is to evaluate the cross correlation of the functions. Numerically this can easily be achieved by calculating the dot product of the two functions. Again, in order to obtain fitness within the convenient interval of [0,1] we need to normalize the product. The cross-correlation fitness *f_cc_* can be then written in the following way,

(7) $$f_{cc}=\left(P\;\cdot \;Q\right)/max\left[P\;\cdot \;P,Q\;\cdot \;Q\right]$$

where · denotes the dot product.

(8) $$P\;\cdot \;Q\;=\;\overset{\text{length}\left(P\right)}{\underset{i=1}{\sum }}P_{i}\;.\;Q_{i}$$

Although the cross correlation provides a rougher estimation of the similarity between two curves than the Hausdorff distance, it is much less *CPU* expensive. Therefore as long as it provides an advantage to the performance, the *f_cc_* fitness will be preferred to the more precise but more expensive Hausdorff metric. A small-scale comparison study between the two fitnesses will be shown in the Results section.

### 3.4. The Main GA Loop

Once an initial population has been created, the following procedure is applied:

(**A**) **Fitness Calculation**. The fitness of the population is calculated. This is done in two steps. First, for each chromosome the corresponding geometric trajectory is calculated, and the magnetic fields that would be measured by each sensor are simulated. Then the normalized cross-products of the experimental data and the simulated data are calculated. As previously stated, the cross products gage the superposition between the experimental and the simulation data. 

Subsequently, two non-standard procedures are applied. First, ten chromosomes with the highest fitness values are separated: they will not be affected by the rest of the procedure until the next generation. They will participate in the selection and exploration operations, but their presence in the next generation is already guaranteed. This process assures that the best fitness can only be increased with the generation’s progress. The average value of those ten chromosomes is called the best fitness of the generation and is noted$\;\tilde{f}$.

The second procedure has been introduced mainly to save running time: It is rather obvious that the population size has a direct effect on the performance of the *GA*. Using few individuals will make the calculation faster but then the convergence may be very slow because of a poor exploration of the search-domain. On the other hand, a large population will certainly cover the search domain, but the calculation *per generation* can be very expensive. The idea is to dynamically adapt the population size to the convergence state of the algorithm. At the beginning of the process a large number of individuals are required in order to correctly scan the optimization search domain. However, as the convergence progresses the search-domain volume naturally decreases. Therefore, when$\;\tilde{f}$ exceeds a given threshold, the size of the population can be reduced by removing a given amount of “worst” chromosomes. This will increase the speed without altering the quality of the space exploration. The number of removed individuals may also depend on$\;\tilde{f}$.

Both thresholds and size reduction values can be adapted to a specific problem.

Once the fitness of the population has been calculated and if at least 10 successive generations have already been created, a stopping condition is checked according to one of the following criteria:

- The generation index *n* exceeds the maximum allowed number *N_max_*.
- The best fitness $\tilde{f}\left(n\right)$ exceeds a threshold value. Beyond this point no significant improvement of the results can be observed. Typical values range between 0.85 and 0.9.
- The best fitness $\tilde{f}\left(n\right)$ has not changed significantly during the last past ten generations. The best fitness change $d\tilde{f}\left(n\right)\;$is calculated according to the formula:
  (9) $$\text{d}\tilde{f}\left(n\right)=100\;\frac{\sum_{j=n-9}^{n}\left[\tilde{f}\left(j\right)-\tilde{f}\left(j-1\right)\right]}{\sum_{j=n-9}^{n}\left[\tilde{f}\left(j\right)\right]}$$

If $d\tilde{f}\left(n\right)\;$is less or equals to a given threshold then the loop exits. If none of the three criteria is fulfilled the calculations continues.

(**B**) **A wheel of fortune**. A wheel of fortune selection routine is used to select the best chromosomes according to a probability proportional to their fitness values [8]. In this selection rule one associate to each chromosome a segment of which the length is proportional to its fitness. These segments are concatenated on a line normalized from 0 to 1. This procedure imitates the mechanism of spinning a casino wheel of fortune by using a linear structure scheme: a random number is picked in the [0,1] interval; this number indicates which chromosome will be selected. In this method “good” chromosomes can be duplicated and “bad” chromosomes can be removed. In order to optimize the selection we have chosen to perform an exponential scaling of the fitness before the wheel of fortune selection [34]. The scaling reduces or amplifies the gaps between the individuals. It is defined by:

(10) $$f_{s}=f_{cc}^{k\left(n\right)}$$

where *f_cc_* and *f*_s_ are the fitness before and after scaling, respectively, and *n* is the current generation. For *k* values close to zero the gaps are reduced and no specific chromosome is preferred. The *GA* performs as a random search algorithm and allows a rather uniform exploration of the search-domain variables. For *k* close to 1 the scaling does not really operate. For *k* larger than 1 the gaps are amplified and only good chromosomes will survive the selection process. We use a simple formula for *k*(*n*) that generates small values of *k* at the early beginning of the process, large values at the end and stays neutral for most generations in between. It is given by:

(11) $$k\left(n\right)={\left(tan\left[\left(\frac{n}{N_{max}+1}\right)\frac{\pi }{2}\right]\right)}^{0.1}$$

where *N_max_* is the maximum allowed number of generations. 

(**C**) **Cross-over**. A cross-over procedure is then applied to the new population. The purpose of the cross-over is to increase the diversity of the population by manipulating the structure of the chromosomes. The chromosomes to which crossover is applied are sorted using a hyper-geometric distribution. Basically it is similar to the binomial distribution but without replacement. The threshold probability is 0.7 to 0.9 meaning that approximately 70% to 90% of the population participates in the cross over process. The number of changes from one generation to the next adds flexibility to the solution process.

For each pair of chromosomes (the “parents”) a cutting point on the gene chain is uniformly and randomly selected. The parents are sliced at the crossing point, defining a “head” and a “tail” for each before and after the crossing point. Then the heads and tails of the two parents are interchanged giving birth to two new chromosomes, the “children”. 

There are now several possibilities: either the children are accepted with no condition, or their acceptation depends on their fitness. For instance, if their fitness is larger than those of their parents the children are accepted. If not, they can be either simply rejected or accepted according to some probability. By making this probability a function of the current generation one adds a simulated annealing modulation to the crossover process [10]. Although this modification usually improves the performance of the *GA* and such improvement may be necessary in some applications, the simulated annealing appears to be rather time consuming. This is because it requires an extra calculation of the fitness of a large part of the population for each generation. 

(**D**) **Mutation**. A mutation operator is now applied. The mutation operator has been proven to give to the *GA* the ergodicity property in the exploration of the search-domain. In other words, mutation allows the *GA* to reach any point in the search-domain without applying a selection rule or crossover. 

Theoretically, mutations could have even been the unique operator that changes the population from one generation to another. However, the time required for convergence would then be extremely long. Similar to the crossover operator the mutating chromosomes are chosen using a hyper-geometric distribution. A common threshold probability can be selected in the range of 0.05 to 0.2, meaning that from 5% to 20% of the population undergo mutation. The ratio of one to five between mutation and crossover is not exceptional in *GA*s. It reflects the natural fact that most individuals of a population do indeed reproduce and change their genetic material by crossing over their chromosomes, while mutation is a rather rare event in the evolution process, and “good” mutation is even less probable. 

In our *GA* we assure good mutation by using an adaptive mutation operator. For each of these chromosomes one or more mutating genes are randomly and uniformly selected. Their new values are obtained by adding a Gaussian noise to the original value. The Gaussian noise is chosen to be the standard deviation of the difference between the value of this gene on the best chromosome and the values of the rest of the population. In this approach, mutation is no longer a random process but it tends to reduce the gap between the “bad” and the “best” individuals while following the evolution of the best chromosomes towards the convergence point. 

(**E**) **Reinsertion of the Best Chromosomes**. Finally the ten best chromosomes that were not affected by crossover and mutations are reinserted into the population by replacing the ten worst chromosomes.

(**F**) **Return to Step A.**

### 3.5. Implementation

The algorithm was first implemented using Matlab^®^. The algorithm running time was approximately 50 s. To improve the CPU time of the algorithm it was coded and compiled by a Microsoft Visual studio^®^ C++ compiler. The compiled algorithm was executed on an Intel Core i7 2670QM CPU@2.2 GHz processor with 8 GB RAM. The process ran using only a single core. A complete *GA* run takes up to 6 s depending on the number of generations that is required by the fitness function to converge.

Furthermore, because the fitness calculation of each chromosome is an independent procedure, it can be processed using different cores. Hence, parallel programming can be performed and can take advantage of the multiple core architecture of the CPU. 

## 4. Results and Discussion

### 4.1. Hausdorff versus Cross-Correlation Fitness

**Figure 2:**
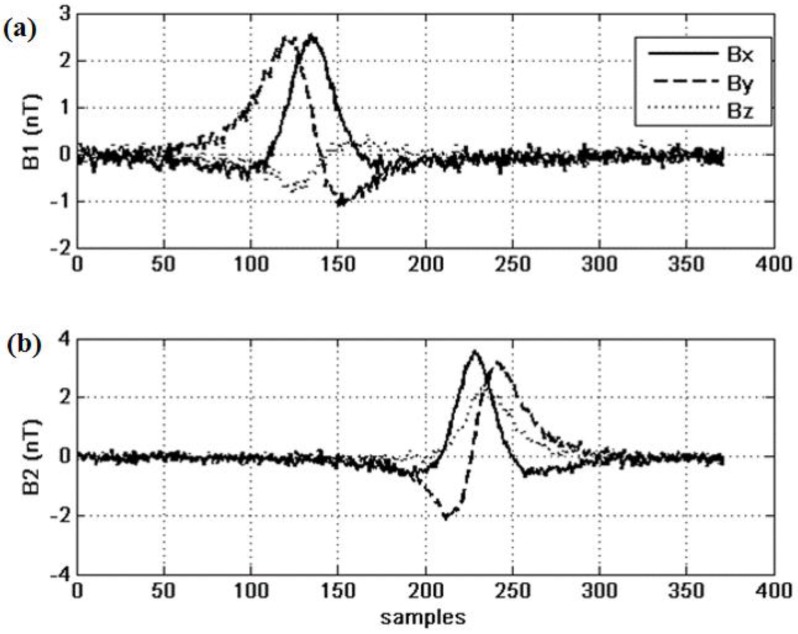
Raw data from the two sensors S1 (**a**) and S2 (**b**).

As presented in the last section, the calculation of the fitness is critical to the success of the GA. Let us consider the Hausdorff distance *versus* the cross-correlation function. Due to running time considerations we consider here a simplified version of the original Hausdorff distance. The system under investigation is the crossing between two sensors S1 and S2. We simulate a trajectory of a ferromagnetic mass with a moment equaling 1 Am^2^, distributed non-homogeneously on the three axis. The path starts at X_0_ = 20 m and crosses the X-axis at equal distance from the center with an increment of 5° (see Figure 1). Fourteen paths are built that differ by their crossing angle: from 10° to 80° with an increment of 5°. For each path we compute the magnetic fields recorded by the sensor to which we add a random Gaussian noise of standard deviation of 0.1 nT. The fields are shown in Figure 2 for a crossing angle of 30°.

**Figure 3:**
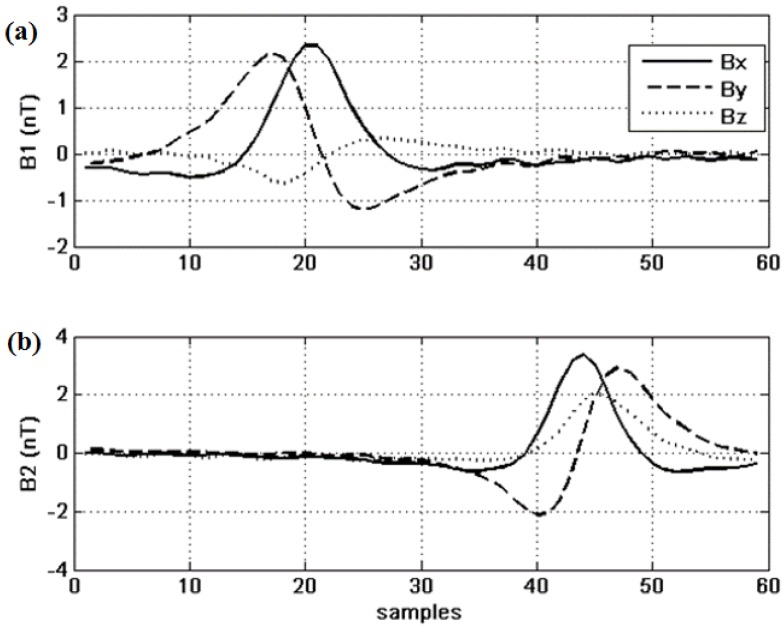
Pre-processed data from the two sensors S1 (**a**) and S2 (**b**) after filttration, detrending and decimation.

The signals are filtered, detrended and decimated; the results are shown in Figure 3. 

**Figure 4:**
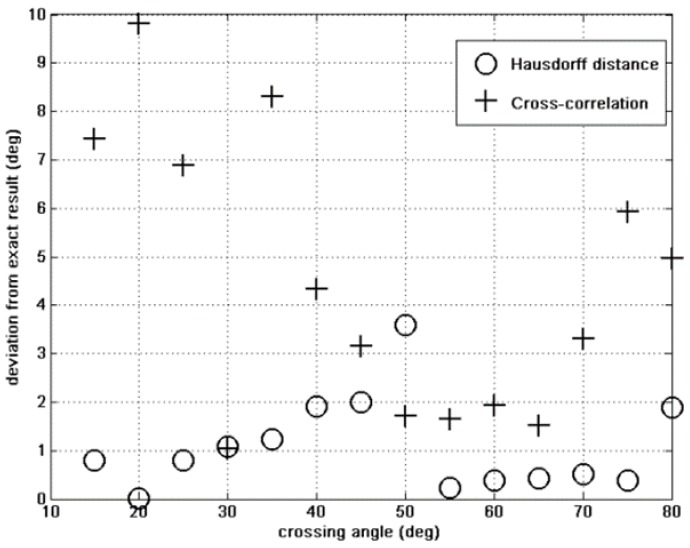
Crossing angle error: Hausdorff *vs.* cross correlation fitness.

**Figure 5:**
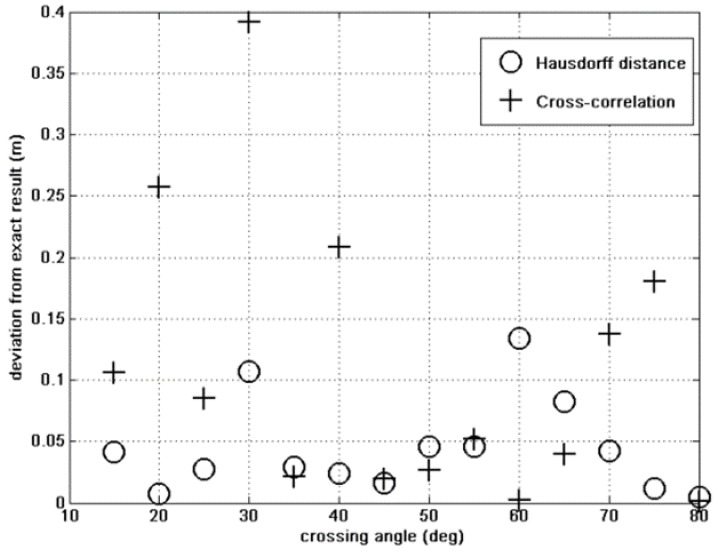
Crossing distance error: Hausdorff *vs.* cross correlation fitness.

**Figure 6:**
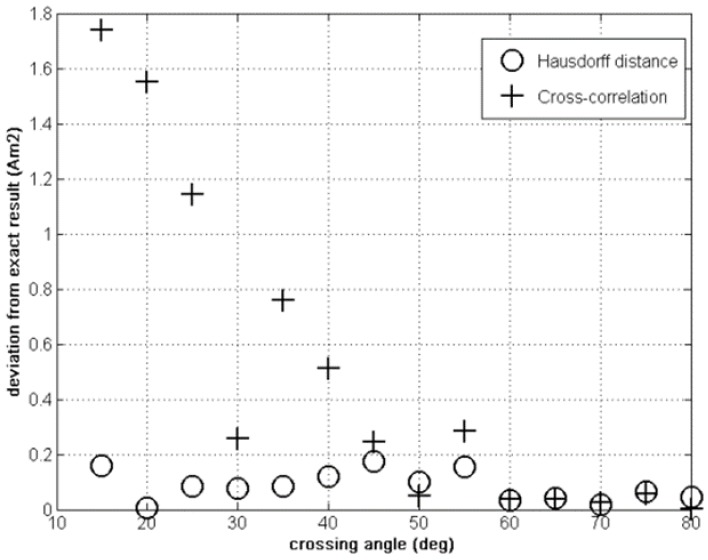
Magnetic moment error: Hausdorff *vs.* cross correlation fitness.

These are the curves that are to be fitted by the GA. The initial population includes 500 individuals, and the state-space is the one we have defined in Section II above. The results of this parameter study are shown in Figure 4, Figure 5 and Figure 6.

Figure 4, Figure 5 and Figure 6 show the finding of the GA for the crossing angle, crossing point and the total magnetic moment. In each figure we can see the results given by the Hausdorff fitness (circles) compared to the cross-correlation fitness (crosses) method. For all variables the Hausdorff distance provides better results than the cross correlation. This was anticipated because the similarity between two curves, which is the basis of the fitness criterion, is better expressed using topology arguments rather than a simple analysis.

In Figure 5 and Figure 6 the cross correlation wins by approximately one order of magnitude. 

**Figure 7:**
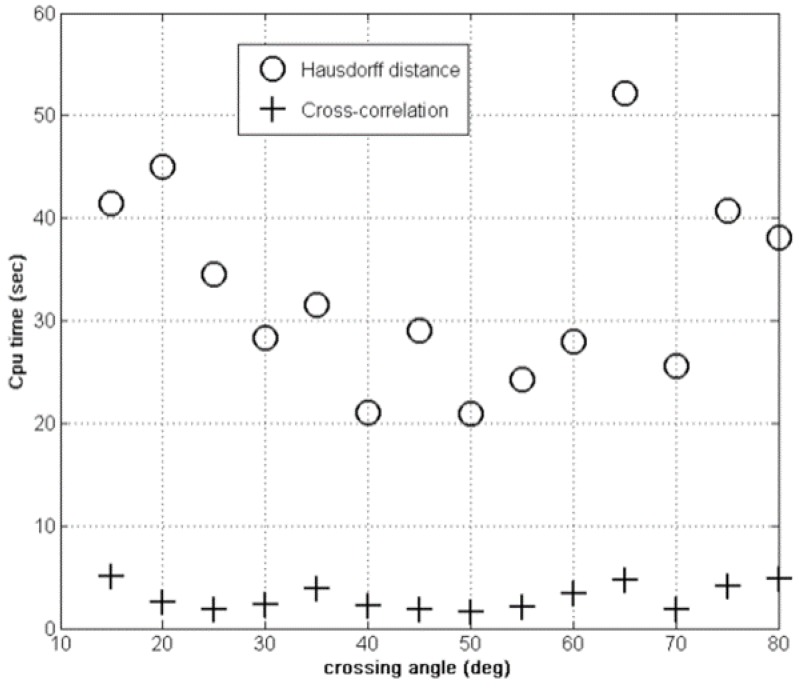
CPU cost: Hausdorff *vs.* cross correlation fitness.

Let us now look at the *CPU* time required by the two methods. This is illustrated in Figure 7. The cross correlation is a better fit for our application because it consumes less CPU time than the *Hausdorff* method while obtaining good accuracy.

### 4.2. Results

We use the *GA* described above in the analysis of a series of real-life (*i.e.*, not simulated) experiments. In these experiments the crossing angle could lie between 0° and 90°. The total moment of the moving object could be large (2 Am^2^), medium (1 Am^2^) or small (0.5 Am^2^). The crossing point was taken at various distances from the two sensors. The velocity was varied between slow (0.5 m/s), medium (1 m/s) and fast (1.5 m/s). Both directions were considered.

**Figure 8:**
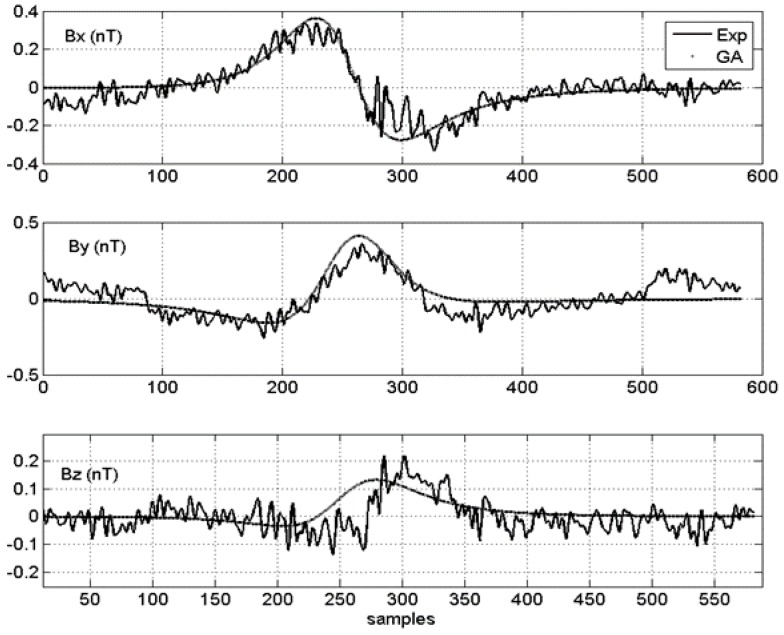
Noisy signal (Signal to noise ratio (SNR) 1): Experimental *vs.* GA solution.

**Figure 9:**
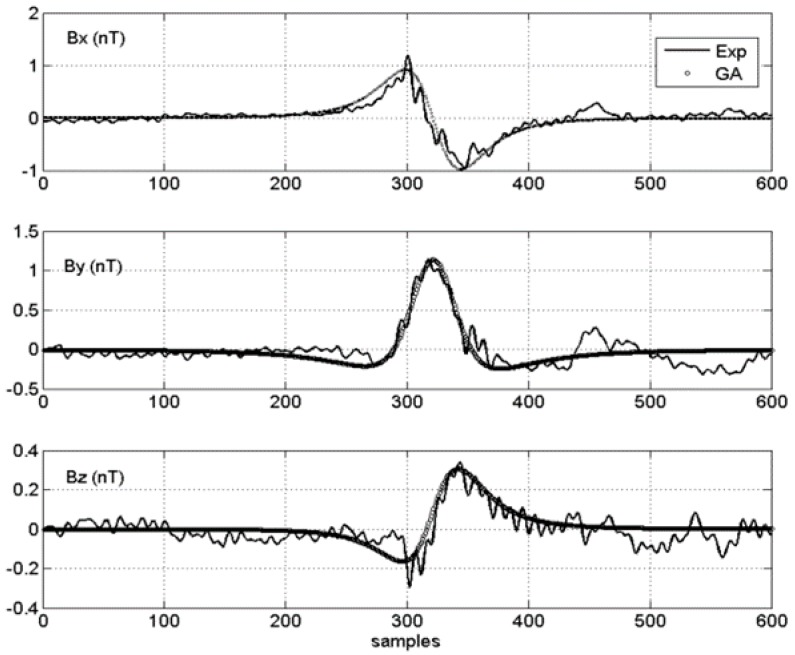
Clean signal (SNR 1): Experimental *vs.* *GA* solution.

The two experiments shown in Figure 8 and Figure 9 differ only in their relative signal to noise ratio (SNR). 

For convenience, if the *SNR* of the noisier signal (Figure 8) is taken as 1, then the *SNR* of the clean signal was estimated to be five, which is higher and better (Figure 9). Each figure shows, for each vector field component, the comparison between the experimental data (thin line) and the field data generated by the best chromosome in the *GA* simulation (thick line). Table 1 summarizes the main findings and differences between the two test cases. 

**Table 1:** Specific points in the cumulative distribution function of the discrepancy between the true crossing point and the *GA* result (in %).

SNR	# of Generations	Best Fitness (%)	Angle Offset (%)	Cross. Dist. Offset (m)	Moment Offset (mA^2^)
1	164	65	18	2	0.2
5	85	80	3	0.8	0.1

**Figure 10:**
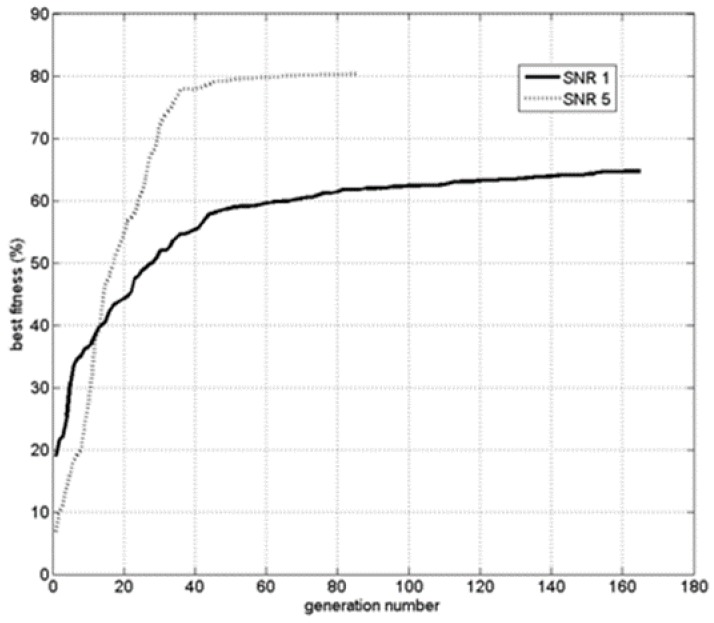
Best fitness value as a function of the iteration number.

The number of generations is the number of iterations leading to algorithm convergence in terms of best fitness stability, as shown in Figure 10. The angle and crossing distance offset expresses the difference between the true value and the corresponding gene in the best chromosome found by the *GA*. The same thing is true for the magnetic moment offset (total field value). As we see even with a relatively low *SNR* the *GA* is able to localize quite precisely the motion and the size of the magnetic object. The best fitness value can serve as a quality index of the result. It can provide an indication of the relative signal to noise ratio of the measured data.

**Figure 11:**
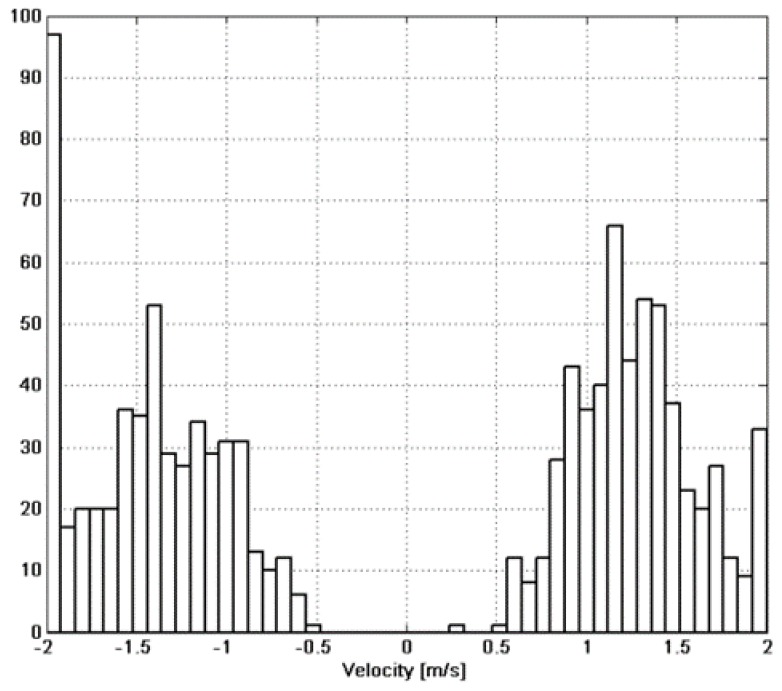
Velocity distribution (*GA* results) for all performed experiments.

Another important characteristic of the path is the object’s velocity. This parameter contains both amplitude (speed) and a sign (direction). The speed was not fully controlled in the experiment but was varied from relatively slow (around 0.8 m/s) to medium (1.1 m/s) and fast (1.5 m/s). Figure 11 shows the speed distribution for all the experiments. The direction of the path was changed alternatively between two crossings in such a way that the sign should change from positive to negative from path to path.

## 5. Conclusions

An upgraded Genetic Algorithm scheme has been developed in order to track moving magnetic objects. It provides an accurate and fast solution to the magnetic localization problem. The scheme has been tested on a full-scale sensor array and the algorithm was validated using real-world experimental data. The algorithm is stable, quick and provides an accurate estimation of all the parameters involved in the various scenarios under investigation. It is now possible to incorporate this GA into localization processes not only for magnetic detection but also for accurate localization. 

## References

[1] Merlat L., Naz P. (2003). Magnetic localization and identification of vehicles. Proc. SPIE.

[2] Salem A., Hamada T., Asahina J.K., Ushijima K. (2005). Detection of unexploded ordnance (UXO) using marine magnetic gradiometer data. Explor. Geophys..

[3] McAulay A. (1977). Computerized model demonstrating magnetic submarine localization. IEEE Trans. Aerosp. Electron. Syst..

[4] Alimi R., Geron N., Weiss E., Ram-Cohen T. (2009). Ferromagnetic mass localization in check point configuration using a Levenberg Marquardt algorithm. Sensors.

[5] Levenberg K. (1944). A method for the solution of certain non–linear problems in least squares. Q. J. Appl. Math..

[6] Marquardt D.W. (1963). An Algorithm for Least-Squares Estimation of Nonlinear Parameters. J. Soc. Ind. Appl. Math..

[7] Dorigo M., Gambardella L.M. (1997). Ant colony system: a cooperative learning approach to the traveling salesman problem. IEEE Trans. Evol. Comput..

[8] Goldberg D.E. (1989). Genetic Algorithms in Search, Optimization, and Machine Learning.

[9] Michalewicz Z. (1996). Genetic Algorithms + Data Structures = Evolution Programs.

[10] Mahfoud S.W., Goldberg D.E. (1995). Parallel recombinative simulated annealing: A genetic algorithm. Parallel Comput..

[11] Min H., Smolinski T.G., Boratyn G.M. A Genetic Algorithm-Based Data Mining Approach to Profiling the Adopters and Non-Adopters of E-Purchasing. ci.uofl.edu/rork/knowledge/publications/min_iri01.pdf.

[12] Lohn J.D., Colombano S.P. (1999). A circuit representation technique for automated circuit design. IEEE Trans. Evol. Comput..

[13] Alander J.T. (2012). An Indexed Bibliography of Genetic Algorithms in Machine Learning.

[14] Levine D. (1996). Application of a hybrid genetic algorithm to airline crew scheduling. Comput. Oper. Res..

[15] Deaven D.M., Ho K.M. (1995). Molecular geometry optimization with a genetic algorithm. Phys. Rev. Lett..

[16] Cunha A.G., Covas J.A., Oliveira P. (1998). Optimization of polymer extrusion with genetic algorithms. IMA J. Manag. Math..

[17] Kundu S., Seto K., Sugino S. Genetic algorithm application to vibration control of tall flexible structures. Electronic Design, Test and Applications, 2002, Proceedings of the 1st IEEE International Workshop.

[18] Chen K.C., Hsieh I., Wang C.A. (2003). A Genetic Algorithm for Minimum Tetrahedralization of a Convex Polyhedron. Ph.D. Thesis.

[19] Mahfoud S., Mani G. (1996). Financial forecasting using genetic algorithms. Appl. Artif. Intell..

[20] Yuret D., Maza M. (1994). A genetic algorithm system for predicting the OEX. Tech. Anal. Stock. Commod..

[21] Smigrodzki R., Goertzel B., Pennachin C., Coelho L., Prosdocimi F., Parker W.D. (2005). Genetic algorithm for analysis of mutations in Parkinson’s disease. Artif. Intell. Med..

[22] Yan H., Jiang Y., Zheng J., Peng C., Xiao S. Discovering Critical Diagnostic Features for Heart Diseases with a Hybrid Genetic Algorithm. Proceedings of the International Conference on Mathematics and Engineering Techniques in Medicine and Biological Sciences, METMBS 2003.

[23] Vinterbo S., Ohno-Machado L. (2000). A genetic algorithm approach to multi-disorder diagnosis. Artif. Intell. Med..

[24] Sharples N.P. (2003). Evolutionary Approaches to Adaptive Protocol Design. Ph.D. Thesis.

[25] Kumar A., Pathak R.M., Gupta M.C. Genetic algorithm based approach for designing computer network topology. Proceedings of the 1993 ACM conference on Computer science.

[26] Noshadi A., Shi J., Lee W.S., Shi P., Kalam A. Genetic algorithm-based system identification of active magnetic bearing system: A frequency-domain approach. Control & Automation (ICCA), Proceedings of the 11th IEEE International Conference.

[27] Noshadi A., Shi J., Lee W.S., Shi P., Kalam A. (2015). Optimal PID-type fuzzy logic controller for a multi-input multi-output active magnetic bearing system. Neural Comput. Appl..

[28] Zolfagharian A., Noshadi A., Khosravani M.R., Zain M.Z.M. (2014). Unwanted noise and vibration control using finite element analysis and artificial intelligence. Appl. Math. Model..

[29] Wynn W.M. (1999). Detection, localization, and characterization of static magnetic dipole sources. Detection and Identification of Visually Obscured Targets.

[30] Costa M.C., Cauffet G., Coulomb J.-L., Bongiraud J.-P., le Thiec P. Localization and Identification of a Magnetic Dipole by the Application of Genetic Algorithms. Proceedings of Workshop on Optimization and Inverse Problems in Electromagnetism-OIPE 2000.

[31] Ginzburg B., Frumkis L., Kaplan B.Z., Sheinker A., Salomonski N. (2008). Investigation of advanced data processing technique in magnetic anomaly detection systems. Int. J. Smart Sens. Intell. Ststems.

[32] Rao K.R., Yip P., Rao K.R. (1990). Discrete Cosine Transform: Algorithms, Advantages, Applications.

[33] Prescott E.C., Rodrick R.J. (1997). Postwar U.S. Business Cycles: An Empirical Investigation. J. Money. Credit. Bank..

[34] Nelson A.L., Barlow G.J., Doitsidis L. (2009). Fitness functions in evolutionary robotics: A survey and analysis. Rob. Auton. Syst..

[35] Aronov B., Har-Peled S., Knauer C., Wang Y., Wenk C. (2006). Fréchet distance for curves, revisited. Algorithms–ESA 2006.

[36] Gómez A., Polo C., Vázquez M., Chen D. (1993). Directionally alternating domain wall propagation in bistable amorphous wires. Appl. Phys. Lett..

